# Azithromycin for Malaria?

**DOI:** 10.4269/ajtmh.16-0332

**Published:** 2016-07-06

**Authors:** Philip J. Rosenthal

**Affiliations:** ^1^ Department of Medicine, University of California, San Francisco, California.

Malaria continues to be one of the greatest infectious disease problems in the world. Antimalarial drugs play an essential role in the treatment and control of malaria. For treatment, older drugs are limited by resistance, but artemisinin-based combination therapy remains highly effective in most areas. However, artemisinin resistance has emerged in southeast Asia,^1^ and resistance to artemisinin partner drugs is already common in many areas.^2^ In Cambodia, where resistance to both artemisinins and piperaquine is prevalent, frequent failures after treatment with dihydroartemisinin–piperaquine have been seen.^3^ We can anticipate that artemisinin resistance will spread to other areas, and that resistance to artemisinins and partner drugs will seriously threaten our ability to treat malaria.

Chemoprevention is an important strategy for malaria control. Nonimmune travelers to malaria-endemic countries are typically prescribed atovaquone–proguanil (Malarone), mefloquine, or doxycycline to prevent malaria. This practice is highly effective, but impractical for endemic populations due to cost and toxicity concerns. In Africa, intermittent preventive therapy is advocated in high-risk populations, with intermittent administration of sulfadoxine–pyrimethamine (SP) to pregnant women, and seasonal administration of SP–amodiaquine to children in the Sahel subregion, where there is a relatively low level of resistance to these drugs. However, the utility of drugs to prevent malaria in endemic populations is limited by resistance to available agents. Monthly dihydroartemisinin–piperaquine has shown strong protective efficacy in African children in some trials,^4^ but is not standard practice yet.

For both treatment and chemoprevention, antimalarial drugs are increasingly limited by resistance. New drugs are greatly needed, and a quite robust pipeline of drugs is under development.^5^ However, development is challenging, typically with slow progress even after promising agents show excellent efficacy, and with the potential for lead compounds to fail in later stages of development. Indeed, no new classes of antimalarial drugs have been broadly approved in a few decades, and it remains unclear if the pipeline will satisfy upcoming needs.

With this background, it behooves us to consider repurposing of available antimicrobial drugs to treat malaria. One such drug is azithromycin, a macrolide antibiotic with broad-spectrum activity against gram-positive and atypical bacteria. As is the case with some other antibacterial protein synthesis inhibitors, including doxycycline, azithromycin exerts antimalarial activity by inhibiting function of the apicoplast.^6^,^7^ This action is necessarily slow. After treatment with doxycycline or azithromycin, parasites are killed by pharmacological concentrations of the drug only in the life cycle after treatment is initiated, presumably due to the ability of parasites to survive most of the life cycle without a functional apicoplast. Yet, doxycycline has a role in our antimalarial armamentarium, both for treatment in combination with quinine and for chemoprophylaxis. Azithromycin has advantages over doxycycline, namely a longer half-life, suggesting the possibility of weekly dosing for chemoprophylaxis, acceptability in young children, who should not be treated with doxycycline if possible, and generally better tolerability than doxycycline.

Azithromycin has already been studied as a potential antimalarial agent. It exerts slow, but potent antimalarial activity via action against the apicoplast organelle.^8^ It is the most potent antimalarial macrolide, with mid-nanomolar activity against cultured *Plasmodium falciparum* after prolonged in vitro incubations.^6^ For the treatment of uncomplicated falciparum malaria, artesunate plus azithromycin offered improved efficacy over artesunate monotherapy, but this regimen was inferior to artesunate plus mefloquine.^9^ Similarly, dihydroartemisinin plus azithromycin had good efficacy, but was inferior to dihydroartemisinin plus mefloquine.^10^ Azithromycin plus chloroquine has been extensively studied against falciparum malaria after a trial in India showed the combination to offer excellent efficacy,^11^ but in Malian children azithromycin plus chloroquine was inferior compared with artemether–lumefantrine.^12^ In this issue of the *American Journal of Tropical Medicine and Hygiene*, Phong and colleagues report on a 3-day regimen of artesunate plus azithromycin for the treatment of falciparum malaria in a small number of children and adults in Vietnam; the regimen was well tolerated and had a corrected treatment efficacy of 96.7%.^13^

For the prevention of falciparum malaria, azithromycin had good preventive efficacy in Kenyan^14^ and Indonesian^15^ adults when administered daily, although the preventive efficacy was inferior to that of doxycycline in both trials (protective efficacy in Kenya was 83% for azithromycin versus 93% for doxycycline; in Indonesia 72% versus 96%). In Kenya, azithromycin preventive efficacy was fairly poor when administered weekly (64%). Mass distribution of azithromycin for the control of trachoma was associated with a reduction in malaria parasitemia compared with controls.^16^ Azithromycin plus piperaquine was well tolerated in pregnant Papua New Guinean women,^17^ although preventive efficacy data are not available.

Considering needs for new antimalarials for treatment and prevention and available data, should we consider azithromycin for this purpose? On the plus side, azithromycin is approved around the world and is generally considered safe in children and in pregnancy. Indeed, if used regularly, azithromycin may have benefits beyond malaria. Intermittent administration of azithromycin has played a major role in efforts to eliminate trachoma^18^ and yaws^19^; regular use of chloroquine plus azithromycin to treat malaria in Malawian children was associated with decreased respiratory and gastrointestinal infections compared with a group receiving only chloroquine^20^; azithromycin plus SP given to pregnant women was associated with increased birthweight^21^; and, remarkably, in a randomized trial in Ethiopia, infrequent (quarterly, biannual, or annual) dosing of azithromycin decreased child mortality by half.^22^ On the other hand, azithromycin efficacy for treatment and chemoprevention has typically been somewhat lower than that of comparator regimens. Also, wider use of azithromycin will probably select for drug-resistant bacterial infections. Lastly, use of the drug has been associated in some,^23^ but not other^24^ trials with increased risk of death from cardiovascular causes; this is probably a modest concern for use in malaria, particularly in children, but nonetheless is reason for caution. In a perfect world, azithromycin would probably not be considered further for the treatment or chemoprevention of malaria, as more efficacious and rapid-acting agents are available. However, limitations in efficacy or rate of action may be circumvented in combination regimens. With the continued threat of drug resistance and a sluggish pipeline for new agents, it seems appropriate to continue to study repurposing azithromycin, a tried-and-true antimicrobial drug for other indications, in combination regimens for the treatment and/or prevention of malaria.
